# Human conjunctival epithelial cell responses to platelet-activating factor (PAF): signal transduction and release of proinflammatory cytokines

**Published:** 2009-06-06

**Authors:** Najam A. Sharif, Shouxi Xu, Peggy E. Hellberg, Iok-Hou Pang, Daniel A. Gamache, John M. Yanni

**Affiliations:** Pharmaceutical Products Research, Alcon Research Ltd., Fort Worth, TX

## Abstract

**Purpose:**

The aims of the study were to characterize the signal transduction responses to platelet-activating factor (PAF) and to monitor the downstream effects of PAF on the production of proinflammatory cytokines in human conjunctival epithelial cells (HCECs).

**Methods:**

The generation of inositol phosphates ([^3^H]IPs) from [^3^H]phosphoinositide (PI) hydrolysis and the mobilization of intracellular calcium ([Ca^2+^]_i_) were evaluated using ion exchange chromatography and Fura-2 fluorescence techniques, respectively. The production of the cytokines (interleukin-6 [IL-6], interleukin-8 [IL-8], and granulocyte macrophage colony-stimulating factor [GM-CSF]) from PAF-stimulated HCECs was quantified using specific ELISA assays. Specific PAF antagonists were used to study the pharmacological aspects of PAF actions in HCECs.

**Results:**

PAF (100 nM) maximally stimulated PI turnover in HCECs by 2.3±0.02 fold (n=21) above basal levels and with a potency (EC_50_) of 5.9±1.7 nM (n=4). PAF or its stabilized analog, methyl carbamyl (mc)PAF (EC_50_=0.8 nM), rapidly mobilized [Ca^2+^]_i_, which peaked within 30–60 s and remained elevated for 3 min. PAF (10 nM–1 µM) stimulated the release of the proinflammatory cytokines, IL-6, IL-8, and GM-CSF, 1.4–3.5 fold above basal levels. The effects of PAF (100 nM) on PI turnover and [Ca^2+^]_i_ were potently antagonized by the PAF antagonists, 1-o-hexadecyl-2-o-acetyl–sn-glycero-3-phospho (N,N,N-trimethyl) hexanolamine (IC_50_=0.69 µM; K_i_=38 nM), methyl 2-(phenylthio)ethyl-1,4-dihydro-2,4,6-trimethyl-pyridine-3,5-dicsrboxylate (PCA-42481; IC_50_=0.89 µM; K_i_=50 nM), rac-3-(N-octadecylcarbomoyl)-2-methoxy) propyl-(2-thiazolioethyl) phosphate (CV-3988; IC_50_=13 µM; K_i_=771 nM), and (+/−)-cis-3,5-dimethyl-2-(3-pyridyl)thiazolidin-4-one HCl (SM-10661; IC_50_=14 µM; K_i_=789 nM [n=3 for each antagonist]). PAF-induced production of IL-6, IL-8, and GM-CSF from HCECs was also blocked by these PAF antagonists (IC_50_=4.6– 8.6 µM).

**Conclusions:**

HCECs respond to PAF by generating IPs, mobilizing [Ca^2+^]_i_, and then secreting cytokines into the extracellular medium. These results suggest that HCECs may be key target cells for the PAF released from conjunctival mast cells following ocular allergic reactions. Therefore, HCECs in culture represent suitable in vitro models for the investigation of the role of PAF in human ocular allergic and inflammatory diseases and for the discovery of therapeutically useful PAF antagonists.

## Introduction

Platelet-activating factor (PAF) is an ether phospholipid, which is produced and released by many types of cells including mast cells, neutrophils, eosinophils, macrophages, and basophils following noxious stimulation and/or after initiation of allergic reactions [1-4]. PAF is a potent proinflammatory agent causing platelet aggregation, enhancing histamine and serotonin release with resultant vasodilation and increased vascular permeability, increased eosinophil and neutrophil motility, and degranulation [1-4] leading to edema formation, hyperemia, itching, and pain [1,5]. Based on these symptomatologies, PAF has been implicated in asthma, shock and thrombosis, and in allergic and inflammatory ocular diseases including various forms of conjunctivitis and neovascularization [1,5]. Indeed, in terms of specific ocular effects, PAF has been found in the cornea, iris, ciliary body, and retina and is released into the tear film upon conjunctival provocation [5-7]. Topically applied PAF causes conjunctival edema and raises intraocular pressure (IOP) [8], and intracamerally injected PAF produces an inflammatory reaction with pronounced aqueous flare, corneal edema, and IOP changes [9]. In addition, intracorneal injection of PAF causes a severe chemotactic response in the cornea and the surrounding conjunctiva [9]. PAF that is exogenously added to cultured cells or rabbit corneal organ cultures induces gene expression of cyclooxygenase-2 [10], plasminogen activator [11,12], and matrix metalloproteinases (MMPs) [13]. In the rat eye, PAF receptor mRNA has been detected in the corneal epithelium [14], but little information is available for human conjunctiva on the mRNA or protein of the PAF receptor. While a preliminary report indicated that specific binding sites for PAF were present on rabbit corneal epithelial cells [15], no such information is known for human conjunctival epithelial cells.

Although human ocular allergies involve several different molecular and cellular mechanisms [1-4], the major involvement of conjunctival mast cells in producing and releasing chemical mediators such as PAF, leukotrienes, histamine, and other agents during allergic reactions is not well established [1-3]. While the conjunctival microvasculature and nociceptive nerve-endings may be the predominant target tissues for the direct actions of PAF and histamine, it is not clear if other cells on the ocular surface are also influenced and/or recruited by these primary mediators in the allergic and inflammatory cascade. Human conjunctival epithelial cells (HCECs) may represent potential target cells for mast cell mediators, and we have recently demonstrated that HCECs respond to histamine by generating inositol phosphates, mobilizing intracellular calcium [16,17], and then secreting proinflammatory cytokines like interleukin-6 (IL-6) and interleukin-8 (IL-8) [18].

The aims of the present studies were threefold: 1) to determine if functionally coupled PAF receptors were present on primary cultures of HCECs isolated from multiple donors using [^3^H]phosphoinositide (PI) turnover as an index of receptor activation, 2) to demonstrate possible PAF-induced mobilization of intracellular calcium ([Ca^2+^]_i_) in HCECs, and 3) to investigate whether the activation of the latter signal transduction mechanism in HCECs leads to the release of a variety of proinflammatory cytokines.

## Methods

### Isolation and culture of human conjunctival epithelial cells

The procedures for isolating and culturing human conjunctival epithelial cells (HCECs) from 21 post-mortem human donors were as previously described with minor modifications [18,19]. Briefly, human conjunctival tissue was aseptically dissected (within 8–12 h of death) and transported from eye banks in Dexol^®^ or Optisol^®^ preservation medium (Chiron Ophthalmics, Irvine, CA) in ice. Tissue from different donors was kept separate and rinsed in phosphate buffered saline (PBS). It was then treated with dispase (Collaborative Research, Bedford, MA) at 10 U/ml in 50% Hanks buffered salts and keratinocyte basal medium (KGM; Clonetics, San Diego, CA), containing 0.05 mM calcium, at 4 °C for 24–48 h. Complete KGM was prepared by adding 30 µg/ml of bovine pituitary extract, 0.5 µg/ml hydrocortisone, 0.05 µg/ml amphotericin B and 50 µg/ml gentamicin, 5 µg/ml insulin, 10 µg/ml transferrin, and 0.05 mM calcium chloride. After incubation with dispase, the slightly yellow conjunctival epithelium was removed with a scalpel from the white connective tissue. The epithelium was gently dissociated to generate individual cells and washed in Dulbecco’s modified Eagle medium (DMEM) containing 10% fetal bovine serum using a centrifugation (280x g/10 min)/re-suspension procedure. The cell pellet was re-suspended in a low calcium (0.05 mM) KGM, plated into T75 flasks (collagen type IV coated), and incubated at 37 °C under a humidified atmosphere of 95% air and 5% CO_2_. The medium was changed a day later and then changed every two days thereafter. The cells became confluent in approximately 10 days at which point they were subcultured (passage 1; P1) by rinsing with PBS, incubating in dispase for about 20 min until the cells detached, washing in DMEM by centrifugation, and then plating on collagen-coated 24 well culture plates (Corning/Costar, Cambridge, MA). All subsequent experiments for PI turnover and intracellular calcium mobilization were performed with the P1 cells. For the [Ca^2+^]_i_ experiments, the cells were cultured on sterilized #0 glass coverslips (Biophysica Technologies Inc., Sparks, MD) for three to seven days.

### PAF-induced PI turnover

Phosphoinositide (PI) turnover assays were conducted as follows. Briefly, HCECs were incubated with [^3^H]myo-inositol (1 µCi/0.5 ml; 15–17 Ci/mmol; Amersham, Arlington Heights, IL) in DMEM for 24 h at 37 °C. After this time, the medium was aspirated and the cells were rinsed with 1 ml of warm DMEM and then exposed to PAF or methyl carbamyl (mc)PAF (10 pM–10 µM; Biomol Inc., Plymouth Meeting, PA) in DMEM that contained 10 mM LiCl for 60 min at 37 °C to stimulate the production and accumulation of [^3^H]inositol phosphates ([^3^H]IPs) [16,17]. To determine the potencies of some commercially available PAF antagonists (Biomol Inc.), the latter drugs were added to the cells 30 min before the addition of PAF. The medium was aspirated at the end of the incubation, and the assay stopped with the addition of 1 ml of ice-cold formic acid (0.1 M). After about 15 min, 0.9 ml of the cell lysates were transferred to Econo-columns^®^ (Bio-Rad, Richmond, CA), which contained 1 ml AG1X8 ion-exchange resin in the formate form. The columns were washed with deionized water (10 ml) to remove the free [^3^H]myo-inositol, which was discarded, and the water-soluble [^3^H]IPs were then eluted from the columns using 4 ml of ammonium formate (1.2 M) made in 0.1 M formic acid. A water-accepting scintillation fluid (15 ml) was then added to the eluates, and the [^3^H]IPs were quantified by liquid scintillation spectrometry on a beta counter at about 50% efficiency. The data obtained from these studies were analyzed using a nonlinear, iterative, sigmoidal curve-fitting computer program [16,17] and the appropriate means±SEM calculated for results from several independent experiments and from several cultures of HCECs from different donors. The PAF antagonists used in these and other studies described below included the following: 1-o-hexadecyl-2-o-acetyl –sn-glycero-3-phospho (N,N,N-trimethyl) hexanolamine (abbreviated to hexanolamine); methyl 2-(phenylthio)ethyl-1,4-dihydro-2,4,6-trimethyl-pyridine-3,5-dicsrboxylate (PCA-42481); rac-3-(N-octadecylcarbomoyl)-2-methoxy) propyl-(2-thiazolioethyl) phosphate (CV-3988); and (+/−)-cis-3,5-dimethyl-2-(3-pyridyl)thiazolidin-4-one HCl (SM-10661).

### [Ca^2+^]_i_ mobilization

Intracellular calcium mobilization studies were performed as follows. Briefly, HCECs (passage 1) were cultured on sterilized glass coverslips (#0; Biophysica Technologies Inc.) for three to seven days. On the day of experiment, the cells were loaded with fura-2 acetoxymethyl ester (Fura-2/AM; Molecular Probes Inc., Eugene, OR) by incubating at room temperature for 30 min with Buffer A (125 mM NaC1, 5 mM KC1, 1.8 mM CaC1_2_, 2 mM MgC1_2_, 0.5 mM NaH_2_PO_4_, 5 mM NaHCO_3_, 10 mM glucose, 0.1% BSA, 5 mM Fura-2/AM, 10 mM HEPES, pH 7.2). After the incubation, the coverslip was rinsed twice with Buffer B (Buffer A without BSA or Fura-2/AM) and mounted in an incubation chamber on the mechanical stage of a Nikon Diaphot microscope (Nikon, Garden City, NY). The chamber was filled with 2 ml of Buffer B at room temperature during the experiment. At times indicated, PAF and/or antagonists were added in volumes of 20 µl and were removed by rinsing with 5 rinses of 2 ml each time of Buffer B. Calcium ratio fluorometry was performed using a DeltaScan-4000 ratio fluorescence system (Photon Technology International, South Brunswick, NJ) [20]. The fluorescent dye, Fura-2, within the cells was excited by alternating between 340 nm and 380 nm excitation wavelengths, and the intensity of the intracellular emission fluorescence at 510 nm for each excitation wavelength was monitored in real time by a SIT microscope video camera (model C2400; Hamamatsu Hamamatsu City, Japan). The intracellular Ca^2+^ concentration ([Ca^2+^]_i_) of each cell was calculated from the intensity ratio of fluorescence at the two excitation wavelengths according to the equation of Grynkiewicz et al. [21] and the appropriate mean±SEM calculated for results from several independent experiments using fresh cells from new donors. In some experiments, when the exact concentration of calcium was not critical to the interpretation of the data, only the intensity ratio was presented. In such cases, a higher ratio indicates higher [Ca^2+^]_i_.

### PAF-induced cytokine release

The release of cytokines and their quantification were performed using specific ELISAs as previously described [18]. Briefly, for each experiment, the cells from an individual donor were trypsinized as above and subcultured overnight at a density of 2×10^4^ viable cells/ml per well using fibronectin (FNC)-coated 24 well Primaria^®^ plates (Becton Dickerson Labware, Lincoln Park, NJ). Duplicate or triplicate cultures were set up for each experimental group. The following morning, the medium was replaced with a fresh KGM medium (deficient in hydrocortisone) with or without PAF, and the cultures were incubated at 37 °C for an additional 24 h unless otherwise specified. Supernatants were collected at various times during the stimulation period for time-course experiments, centrifuged at 200x g for 3 min, transferred into separate tubes, and stored frozen at -20 °C. When the effects of PAF antagonists were investigated, they were added to the cells simultaneously with PAF. Cell monolayers were lysed by freeze–thaw cycling at -70 °C followed by sonication on ice. The lysates were clarified by centrifugation as described and stored at -20 °C. The cell culture supernatants were analyzed for IL-6, IL-8, and GM-CSF by ELISA (Quantikine Kits™; R&D Systems, Minneapolis, MN) as directed by the manufacturer. The optical density readings of the resultant color change were measured at 450 nm (540 nm reference) using a Bio-Tek™ microplate reader (Bio-Tek Instruments, Winooski, VT).

### Data analyses

Functional data (dpm/well; pmol/well, etc.) were analyzed using a nonlinear, iterative, sigmoidal curve-fitting computer program (Origin®; OriginLab Corp., Northamptin, MA)  [16,17]. Data from several experiments from different donors were combined and represented as the percentages of the maximal responses/inhibitions to provide an overall summary of the key pharmacological data. The molar potency (EC_50_) values represented the concentration of PAF required to produce 50% of the maximal response. The molar antagonist potency (IC_50_) represented the concentration of the PAF antagonists to cause 50% inhibition of the maximal functional response induced by PAF. Equilibrium drug inhibition constants (drug dissociation constants; K_i_s) derived from the functional assays represented the molar drug concentrations required to produce 50% inhibition of the response while the pK_i_ values represented the –log of this parameter. The equilibrium antagonist potency (K_i_) values were calculated according to the equation [16,18]:

$${\text{K}}_{\text{i}}={\text{IC}}_{\text{5}0}\;/\left[\text{1 }+\;\left({\text{Agonist concentration/Agonist EC}}_{\text{5}0}\right)\right]$$

where IC_50_ is the concentration of the antagonist inhibiting the PAF-induced response by 50%. All data from three or more experiments were calculated as mean±SEM.

## Results

### PI turnover studies

**Figure 1:**
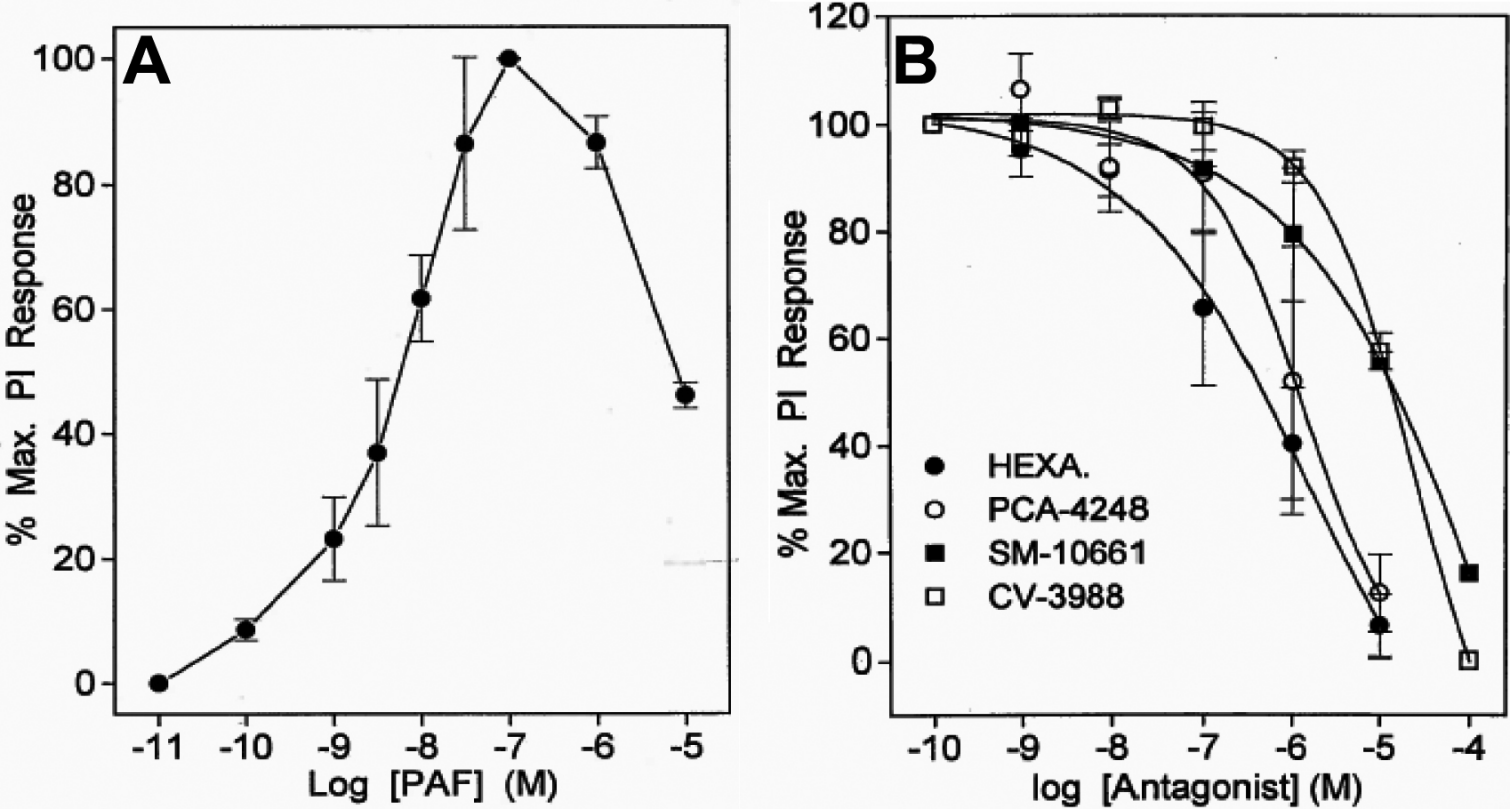
Effect of PAF on PI turnover and blockade of this response by PAF antagonists in HCECs. The concentration-dependency of PAF-induced PI turnover is shown in **A** while the antagonism of PAF (10 nM)-stimulated PI turnover by different concentrations of four PAF antagonists is shown in **B**. Data are presented as mean±SEM from three or more experiments. Hexa=hexanolamine.

PAF (100 nM) maximally stimulated PI turnover in primary HCECs by 2.3±0.02 fold (n=21) above basal levels and with a potency (EC_50_) of 5.9±1.7 nM (n=4; Figure 1A). For example, the [^3^H]IPs generated by 100 nM PAF were 10,160±1,254 dpm (n=21) while the basal control levels of [^3^H]IPs were 4,373±606 dpm (n=21). This represents a 2.3 fold stimulation above basal levels. However, the PI response diminished significantly relative to the maximal response when the concentration of PAF was increased to 1–10 µM (Figure 1A).

The effects of PAF (100 nM) on PI turnover were potently and concentration-dependently blocked by the PAF antagonists, hexanolamine (IC_50_=694 nM; K_i_=38 nM; -log K_i_ [pK_i_]=7.4±0.12), PCA-42481 (IC_50_=897 nM; K_i_=50 nM; pK_i_=7.4±0.23), CV-3988 (IC_50_=13.2 µM; K_i_=771 nM; pK_i_=6.1±0.21), and SM-10661 (IC_50_=14 µM; K_i_=789 nM; pK_i_=6.1±0.01 [n=3 independent experiments for each antagonist]; Figure 1B).

### [Ca^2+^]_i_ mobilization studies

**Figure 2:**
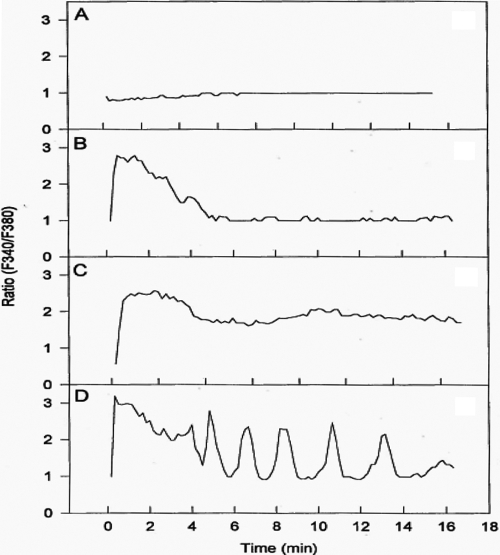
Representative different calcium responses of HCECs to PAF. PAF (10 nM) was added at 0 min. The y-axis shows the intensity ratio of fluorescence at the two excitation wavelengths of a single cell. High ratio indicates higher [Ca^2+^]_i_. **A** depicts a minimal response of a cell to PAF. **B** shows a rapid response to PAF followed by a relatively rapid decay of the [Ca^2+^]_i_ mobilization response. **C** displays a PAF-induced [Ca^2+^]_i_ mobilization response that lasts for at least 16 min. **D** shows an apparent oscillatory [Ca^2+^]_i_ mobilization pattern in an HCEC. The basal control effects with just the vehicle resembled the lack of response in **A**.

**Figure 3:**
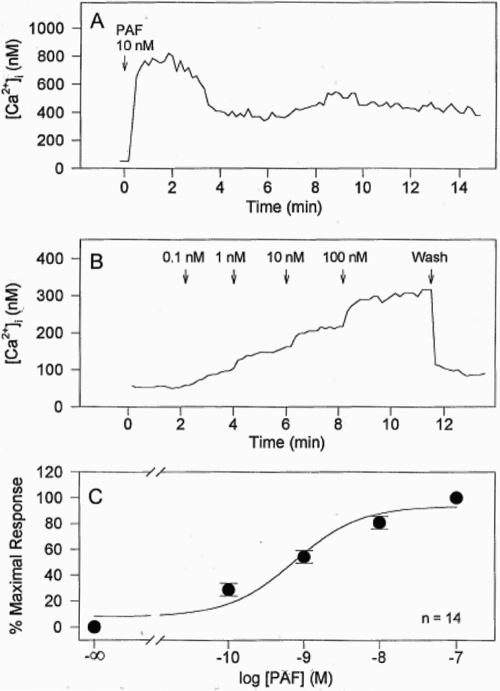
Effects of PAF on [Ca^2+^]_i_ in HCECs. **A** shows representative changes in [Ca^2+^]_i_ of a single cell. **B** shows the effects of adding various concentrations of PAF to HCECs at different time points and the changes in [Ca^2+^]_i_. Wash=drug removal by rinsing the cells with 5 rinses of 2 ml each time with assay buffer. **C** shows the concentration-response curve for PAF obtained by plotting peak calcium responses after PAF treatment versus the corresponding PAF concentrations. Resting intracellular calcium concentration defines 0% response and calcium concentration after 100 nM PAF defines 100% maximum response. In **C**, each symbol (filled circle) represents the mean±SEM (n=6).

PAF was observed to stimulate [Ca^2+^]_i_ mobilization in most cells studied. However, the cellular responses varied for 10 nM PAF, ranging from no response (Figure 2A), a rapid response within a few seconds followed by response decay (Figure 2B), a rapid response followed by sustained [Ca^2+^]_i_ mobilization (Figure 2C), and a rapid response followed by oscillatory behavior (Figure 2D). The predominant type of response, however, was that shown in Figure 2C. The [Ca^2+^]_i_ mobilized in 76 cells peaked after treatment with 10 nM of PAF or mcPAF, which was then followed by a sustained elevated plateau level lasting at least 15 min (Figure 2C; Figure 3A). During the current studies, the mean resting [Ca^2+^]_i_ in primary HCECs was found to be 57±8 nM (mean±SEM, n=23), and this increased to different levels depending on the cell studied and the concentration of PAF added to the incubation medium.

PAF or mcPAF caused an increase in [Ca^2+^]_i_ in most primary HCECs in a concentration-dependent manner (Figure 3B). A detectable increase in [Ca^2+^]_i_ was observed when 0.1 nM of PAF was added to the HCECs, maximal responses to PAF in PI (Figure 1A) and Ca2^+^ (Figure 3B) assays were induced by 100 nM PAF from a concentration-response viewpoint. Cumulative concentration-response curves were constructed for [Ca^2+^]_i_ mobilization by plotting the peak responses versus the corresponding PAF concentrations (e.g., Figure 3C). The mean EC_50_ value for PAF was calculated to be 0.81 nM (-log EC_50_ [pEC_50_]=9.09±0.18, n=14). The mean peak [Ca^2+^]_i_ mobilized by 10 nM PAF was 622±82 nM (n=23), and the mean peak [Ca^2+^]_i_ mobilized by 10 nM mcPAF was 792±140 nM (n=4). These potency values correlated well with the actions of PAF on PI turnover described above. Since the effects of PAF and mcPAF on [Ca^2+^]_i_ mobilization in these cells were essentially identical, data obtained for the two compounds were pooled and analyzed together in the following studies.

Significant desensitization of the PAF-induced [Ca^2+^]_i_ mobilization responses was observed when the cells were treated repeatedly with PAF or mcPAF. Typically, the second treatment with PAF generated only 0%−20% of the response of the first treatment (data not shown). Extensive rinsing of the cells or a long waiting period (up to 30 min) between the agonist additions did not eliminate the desensitization phenomenon. Thus, the same cells were not used for multiple agonist stimulations.

**Figure 4:**
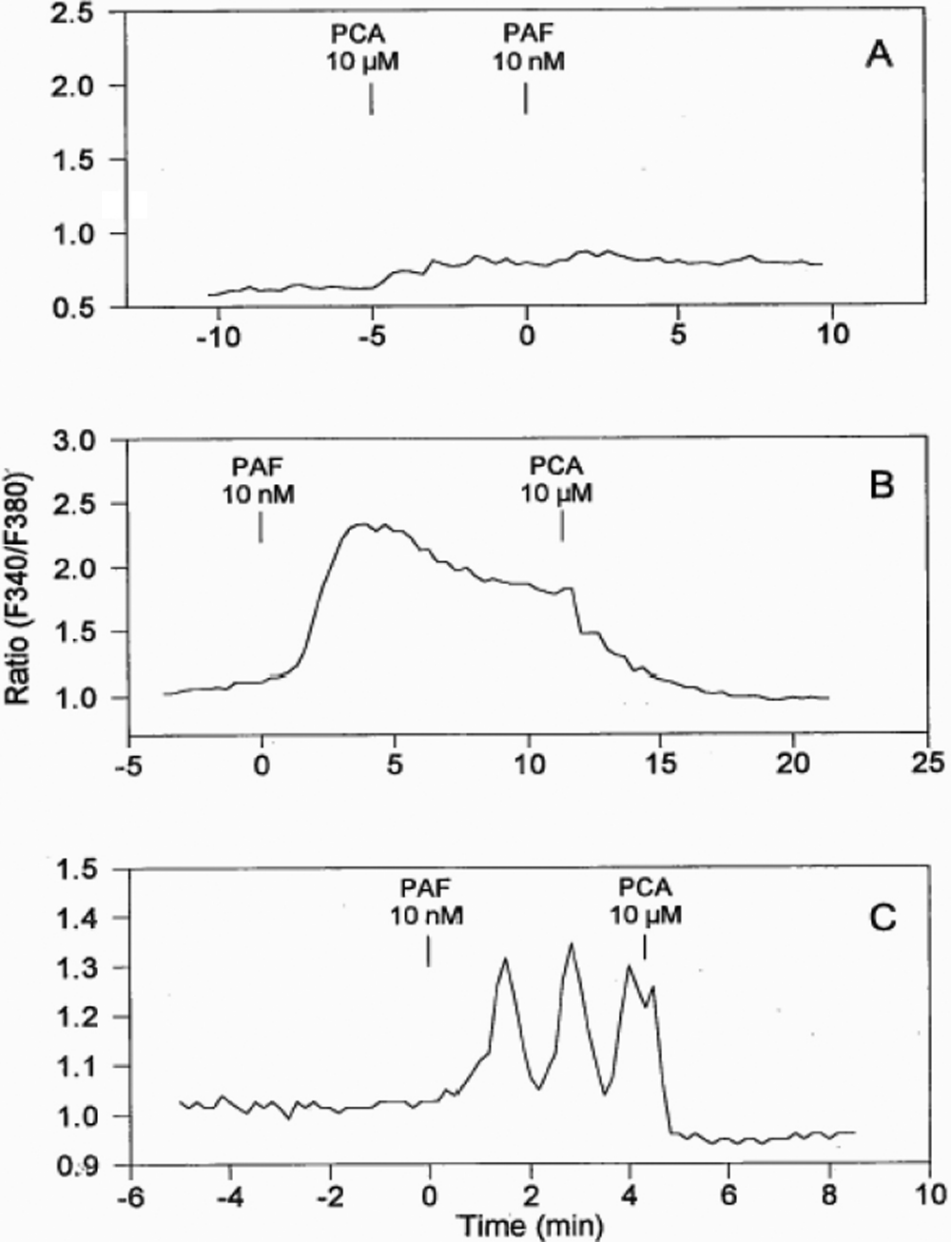
Effect of a PAF antagonist, PCA, on PAF-stimulated [Ca^2+^]_i_ mobilization in HCECs. Tracings of representative singe cells are shown. The y-axis shows the intensity ratio of fluorescence at the two excitation wavelengths of a single cell. Higher ratio indicates higher [Ca^2+^]_i_. Additions of various compounds at various time points are indicated. **A** shows how pretreatment of HCECs with PCA (10 µM) blocked the PAF-induced effect. Similar results were obtained in a total of 25 cells. **B** shows how the PAF-induced elevated [Ca^2+^]_i_ was eliminated immediately upon the addition of PCA. Similar results were obtained in a total of 14 cells. **C** shows how the PAF-induced oscillation of [Ca^2+^]_i_ mobilization was eliminated when PCA was added. Similar results were obtained in a total of seven cells.

To confirm that the [Ca^2+^]_i_ mobilizing effect of PAF was mediated by specific PAF receptors on HCECs, PCA-42481 (a PAF antagonist) was used to antagonize the PAF-induced response. Based on its blockade of PAF-activated PI turnover (Figure 1B), 10 µM PCA-42481 was expected to completely block the action of PAF or mcPAF in the HCECs. Indeed, pretreatment of 25 HCECs with 10 µM PCA-42481 followed by the addition of PAF (10 nM) resulted in minimal mobilization of [Ca^2+^]_i_ (Figure 4A), indicating effective blockade of the PAF receptor and its subsequent coupling to phospholipase C. PCA-42481 effectively and immediately antagonized the PAF-induced [Ca^2+^]_i_ mobilization when added after the initiation of the PAF-induced response in 14 cells (e.g., Figure 4B,C).

**Figure 5:**
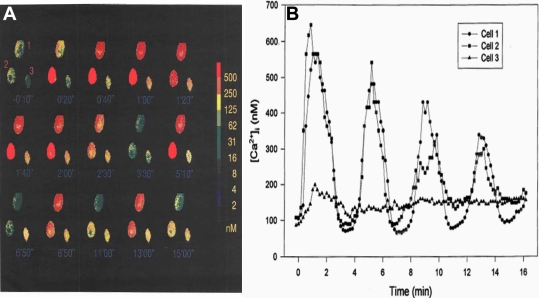
Differential temporal changes in [Ca^2+^]_i_ in HCECs exposed to PAF. **A**: The pseudocolor ratio images of fura-2 loaded HCECs show time-dependent changes of [Ca^2+^]_i_ after 10 nM PAF treatment. PAF was added at time 0. The spectrum on the right indicates a calculated calcium concentration represented by each color. Note that [Ca^2+^]_i_ of cells #1 and #2 oscillated after an initial peak whereas cell #3 mainly had a sustained elevation of calcium concentration during the 15 min recording. **B**: Temporal changes in [Ca^2+^]_i_ in the HCECs exposed to 10 nM PAF are shown. PAF was administered at time 0. The oscillatory nature of [Ca^2+^]_i_ mobilization is readily apparent in cell #1 and #2.

Since initial studies indicated that some HCECs responded to PAF by exhibiting an apparent oscillatory [Ca^2+^]_i_ mobilization pattern (Figure 2D), a more extensive evaluation of this phenomenon was investigated. Figure 5A depicts the temporal changes in [Ca^2+^]_i_ levels, which were visualized in pseudocolor, in three HCECs that were in close proximity of each other when exposed to 10 nM PAF. Followed over time, while cells #1 and #2 showed robust cyclic oscillatory increases and decreases in [Ca^2+^]_i_ (Figure 5B),  cell #3 exhibited a relatively small increase in [Ca^2+^]_i_ at the onset but this declined over time and remained slightly elevated above basal levels (Figure 5B).

### PAF-induced cytokine release

**Figure 6:**
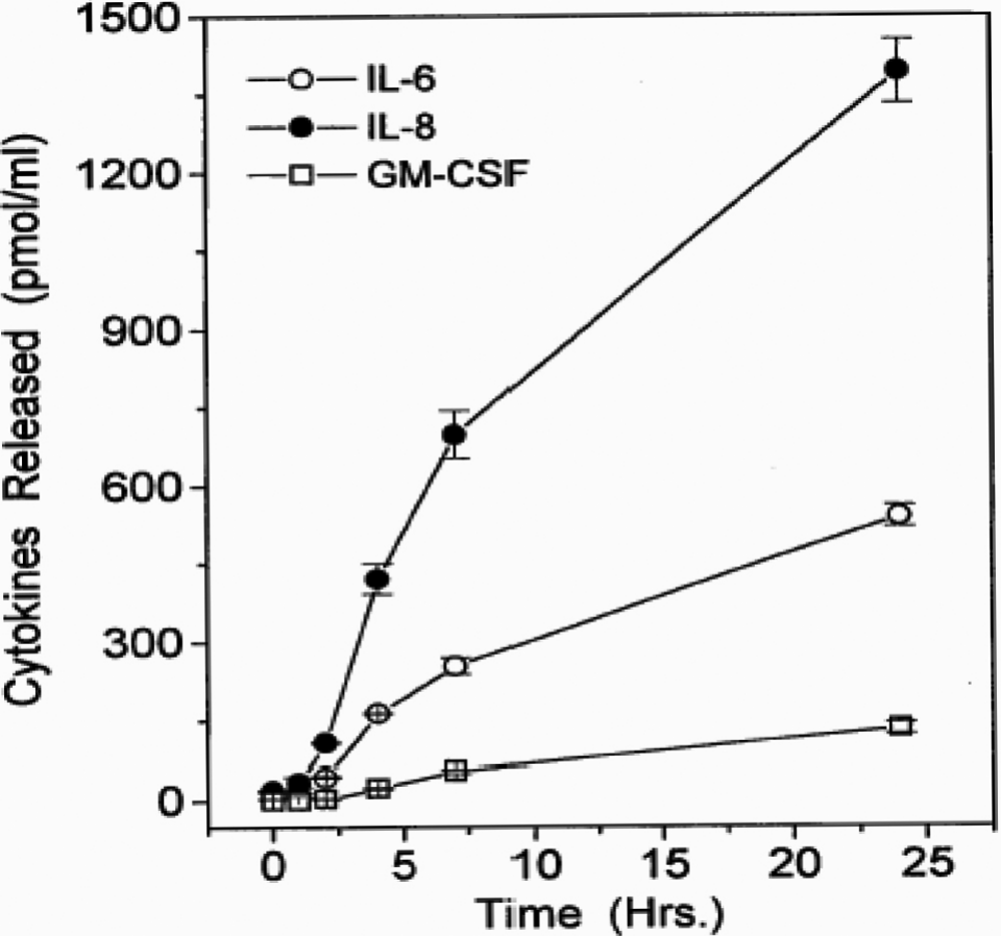
Time-course of cytokine release in HCECs exposed to PAF. The levels of various cytokines (IL-6, IL-8, and GM-CSF) were determined by specific ELISA assays when PAF (1 µM) was added to HCECs isolated from different donor eyes. Data are shown as mean±SEM from three or more experiments. For reference purposes, the basal levels of each cytokine measured were as follows (pg/ml): IL-6=151±33; IL-8=479±77; GM-CSF=44±19.

**Figure 7:**
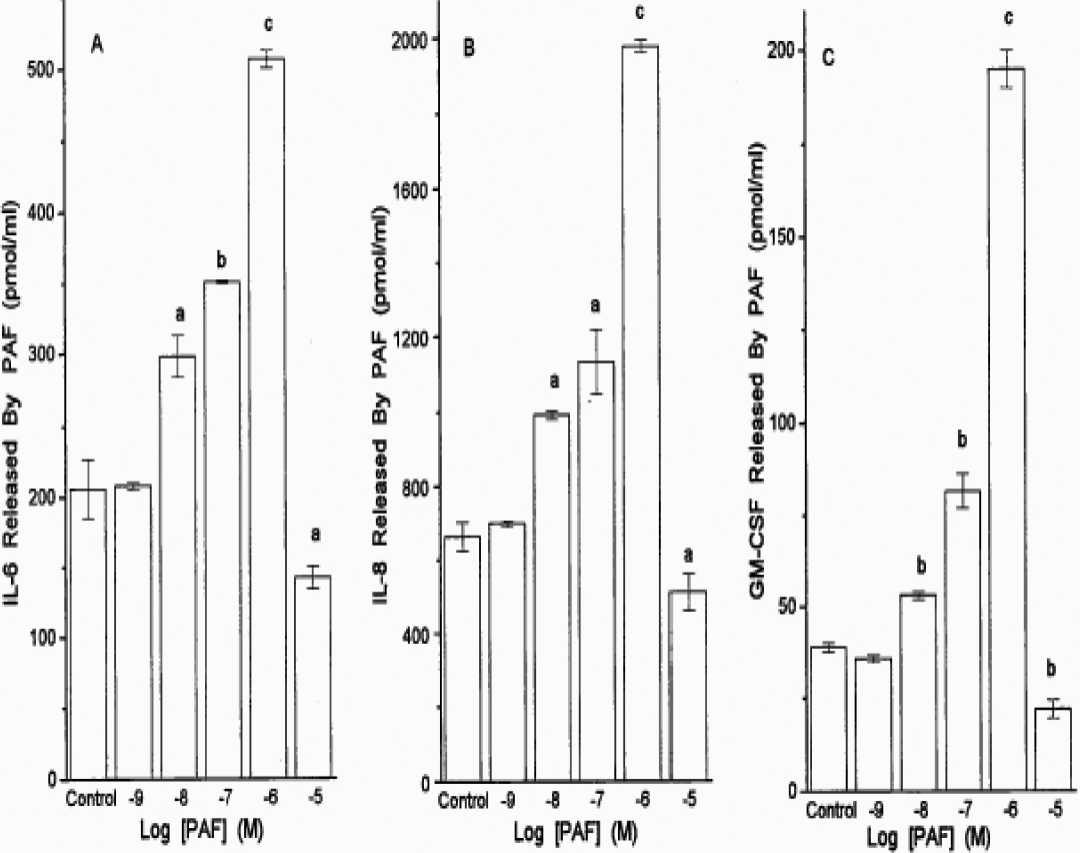
PAF-induced production of various cytokines in HCECs. Concentration-response studies were conducted with PAF in HCECs isolated from different donor eyes, and the concentrations of IL-6 (**A**), IL-8 (**B**), and GM-CSF (**C**) released into the culture medium were determined by specific ELISA assays. The levels of the cytokines produced by buffer alone or by PAF itself are also shown. Data are shown as mean±SEM from three or more experiments. a=p<0.05; b=p<0.01; c=p<0.001, each p value relative to the control.

In a time-dependent manner, PAF significantly increased the release of IL-6 and IL-8. (Figure 6). For IL-6 and IL-8, the first phase of release was linear up to 8 h and the second phase was linear up to 24 h following the addition of PAF (Figure 6). Quantitative determination of cytokines released after 24 h following the addition of PAF yielded the following combined data from several experiments (pg/ml): basal IL-6=151±33, +PAF (1 µM)=415±78; basal IL-8=479±77, +PAF (1 µM)=1403±220; basal GM-CSF=44±19, +PAF (1 µM)=156±42 (1.4–3.5 fold above basal; all mean±SEM from four to six experiments using HCECs from four to six different donors; Figure 7). The estimated potency of PAF in stimulating the secretion of these cytokines using cells from different donors over the 24 h stimulation period was 108±48 nM (n=6; Figure 7).

It was noticeable that treatment of HCECs with 10 µM PAF for 24 h resulted in significantly reduced cytokine release relative to that induced by 10 nM–1 µM PAF (Figure 7). Trypan blue exclusion experiments revealed that the 24 h exposure of HCECs to 10 µM PAF caused a total loss of cell viability, and thus, this concentration of PAF was toxic over this period of stimulation. However, the mechanism of cell death induced by 10 µM PAF was not studied here. This profile of results matched those pertaining to the effects of PAF at different concentrations on PI hydrolysis in HCECs. High concentrations of PAF produce a lower response, presumably due to the toxic effects of PAF (Figure 1).

**Figure 8:**
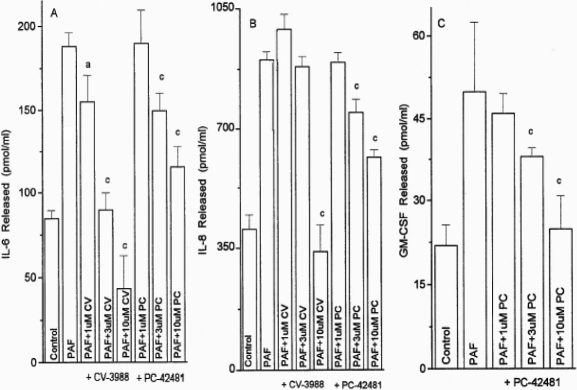
Antagonism of PAF-induced cytokine release in HCECs. Concentration-response studies were conducted with two PAF antagonists (CV-3988 and PC-42481) against a fixed concentration of PAF (1 µM) in HCECs isolated from different donor eyes, and the concentrations of IL-6, IL-8, and GM-CSF released into the culture medium were determined by specific ELISA assays. The levels of the cytokines produced by buffer alone or by PAF itself are also shown. Data are shown as mean±SEM from three or more experiments. a=p<0.05; b=p<0.01, and c=p<0.001, each p value relative to the control. The PAF antagonists by themselves did not influence cytokine release.

The PAF-induced (1 µM) secretion of IL-6, IL-8, and GM-CSF from the HCECs was antagonized by CV-3988 and PCA-42481 in a concentration-dependent (1–10 µM) manner in two separate experiments using HCECs from two different donors (Figure 8). The potencies (IC_50_s) of CV-3988 and PCA-42481 for blocking the PAF-induced cytokine release from HCECs were 4.6–8.6 µM (p<0.05–0.001).

## Discussion

The data presented herein have clearly shown the presence of functionally coupled PAF receptors in primary HCEC cultures isolated from several post-mortem human donor eyes. The utilization of PI turnover, calcium ratio fluorometry, and cytokine ELISA techniques have helped delineate the signal transduction mechanisms and downstream biological events associated with these receptors in these particular cells. Therefore, the PAF receptors on HCECs are positively coupled to phospholipase C, which hydrolyzes membrane phosphoinositides to generate inositol phosphates. Of the latter, the inositol trisphosphate [22] most likely initiated the release of calcium from intracellular stores, which may further increase the influx of extracellular calcium, and cause the subsequent secretion of the cytokines from the HCECs. The fact that the nanomolar potencies of PAF and mcPAF activate all three cellular functions strongly suggests that the biochemical cascade of events mentioned above is closely connected with the pathological roles of the PAF receptors in these cells. These potencies correlate well with the amounts of PAF measured in the tears of ocular allergic patients following conjunctival provocation or in post-alkali burned rabbit eyes [5,10] or in the eyes of sensitized guinea pigs after topical ocular treatment with ovalbumin [7]. Additionally, we demonstrated that all three signal transduction/biological end responses induced by PAF were blocked concentration-dependently by more than three structurally different PAF antagonists (hexanolamine, PCA-42481, CV-3988, and SM-10661). While the predominant effect of PAF in the rabbit cornea appears to be to activate the phospholipase A2/cyclooxygenase pathway [5] and to release leukotrienes that cause conjunctivitis [23], the work presented here has shown that PAF activates the phospholipase C-mediated events in HCECs to release cytokines. However, the activation of the phospholipase A2/cyclooxygenase pathway by PAF in the HCECs cannot be ruled out at this stage and requires further study.

PAF receptor agonists stimulated [Ca^2+^]_i_ mobilization in these cells in a concentration-dependent and antagonist-reversible manner. We have demonstrated previously that histamine also increases [Ca^2+^]_i_ mobilization in these cells [16,17]. However, the time-courses of calcium responses inducted by PAF and histamine were very different. Elevation of [Ca^2+^]_i_ peaked within 5−10 s after histamine treatment while PAF treatment required between 1−2 min for calcium levels to reach its maximum in the responsive cells. This difference may be explained by the fact that PAF analogs have a higher degree of freedom in their molecular structures than histamine. It may take longer for a PAF molecule to reach its optimal conformation for interacting with its receptor, to recruit phospholipase C, and to liberate intracellular inositol phosphates from the membrane phospholipids. The latter hypothesis is also perhaps supported by the fact that HCECs responded uniformly to histamine where it caused a single [Ca^2+^]_i_ peak followed by a sustained elevation of [Ca^2+^]_i_ in almost all cells tested [17] whereas there were at least four different types of cell responses to PAF in the current study.

The multiple responses to PAF in the HCECs are fascinating. As stated above, the cells were morphologically similar under microscopic observation. Our previous publication also shows that they were essentially phenotypically homogenous as indicated by the similar distribution of various selective antigens in all cells, as shown by immunohistochemistry [19]. It is not known at this time if the cells were at different stages of the cell cycle and if that contributes to their different [Ca^2+^]_i_ responses. It is also possible that the cell responses were affected by their microenvironment. For example, a particular class of response may require the presence of a co-factor that is released by neighboring cells. If so, the location of a cell in the culture plate in relation to other cells will determine its response to PAF. Future studies are being planned to address these hypotheses.

Among the four different calcium responses to PAF, the most interesting one was the PAF-induced oscillations of [Ca^2+^]_i_. According to Berridge [22,24], the oscillatory patterns in these cells can be classified as transient oscillations but not sinusoidal oscillations. The properties of these patterns agree with a two pool calcium store model [22,24], whose mathematical representation was presented by Dupont et al. [25]. In this model, an agonist-induced increase in IP_3_ stimulates the release of calcium from an IP_3_-sensitive intracellular calcium storage site into the cytoplasm. Initially, this released calcium is quickly taken up by an IP_3_-insensitive calcium store. However, after such a store is full, the subsequent accumulation of calcium in the cytoplasm can then activate a rapid calcium-induced calcium release from the same IP_3_-insensitive store, which produces the onset of the calcium spike. The recovery of the cytoplasmic calcium occurs with extrusion across the plasma membrane. The continuous presence of the agonist and IP_3_ initiates another cycle of calcium release and thus repetitive spikes and oscillations of cytoplasmic calcium concentration continue. It is presently not clear if these steps were present in the HCECs after PAF treatment. Future studies will endeavor to address this issue.

Exogenously added PAF to HCECs resulted in a time- and concentration-dependent secretion of the proinflammatory cytokines, IL-6, IL-8, and GM-CSF. Unlike the signal cascade events discussed above, several hours were required for the cytokines to be released. This is not unprecedented since it is likely that the upregulation of gene expression and protein synthesis of the cytokines had to occur over a period of time as demonstrated in other cell types and in response to various secretagogues including histamine, calcium ionophore, phorbol ester, and bradykinin [18,26,27]. PAF-induced cytokine synthesis and secretion has the potential to amplify acute inflammatory responses and to lead to the chemosis of neutrophils, basophils, and T-lymphocytes and the potentiation of expression of adhesion molecules, proinflammatory enzymes, and to cause the release of additional cytokines [28-30]. Furthermore, PAF can induce eosinophil accumulation and enhance the expression of PAF receptors, which leads to increased vascular permeability, edema, and itching [31-33].

In summary, PAF potently activates the phospholipase C-coupled signal cascade system to initiate the synthesis and secretion of numerous proinflammatory cytokines in HCECs. Therefore, in addition to the direct deleterious effects of PAF on the ocular surface epithelial cells such as apoptosis [34], corneal remodeling [35], and neovascularization [36], the further amplification of its downstream effects due to the release of cytokines make PAF a powerful inflammatory agent that is implicated in allergic conjunctivitis, pain, and neovascularization of the ocular surface [37-39]. Clearly, PAF receptor antagonists represent useful therapeutic agents to combat ocular surface disorders where PAF may be released into the tear film.
